# Clinically-Driven Virtual Patient Cohorts Generation: An Application to Aorta

**DOI:** 10.3389/fphys.2021.713118

**Published:** 2021-09-01

**Authors:** Pau Romero, Miguel Lozano, Francisco Martínez-Gil, Dolors Serra, Rafael Sebastián, Pablo Lamata, Ignacio García-Fernández

**Affiliations:** ^1^Computational Multiscale Simulation Lab, Department of Computer Science, Universitat de Valencia, Valencia, Spain; ^2^Department of Biomedical Engineering, School of Biomedical Engineering and Imaging Sciences, Kings College London, London, United Kingdom

**Keywords:** virtual cohort, thoracic-aorta, digital twin, synthetic population, clinically-driven sampling, support vector machine, generative adversarial network, *in-silico* trials

## Abstract

The combination of machine learning methods together with computational modeling and simulation of the cardiovascular system brings the possibility of obtaining very valuable information about new therapies or clinical devices through *in-silico* experiments. However, the application of machine learning methods demands access to large cohorts of patients. As an alternative to medical data acquisition and processing, which often requires some degree of manual intervention, the generation of virtual cohorts made of synthetic patients can be automated. However, the generation of a synthetic sample can still be computationally demanding to guarantee that it is clinically meaningful and that it reflects enough inter-patient variability. This paper addresses the problem of generating virtual patient cohorts of thoracic aorta geometries that can be used for *in-silico* trials. In particular, we focus on the problem of generating a cohort of patients that meet a particular clinical criterion, regardless the access to a reference sample of that phenotype. We formalize the problem of clinically-driven sampling and assess several sampling strategies with two goals, sampling efficiency, i.e., that the generated individuals actually belong to the target population, and that the statistical properties of the cohort can be controlled. Our results show that generative adversarial networks can produce reliable, clinically-driven cohorts of thoracic aortas with good efficiency. Moreover, non-linear predictors can serve as an efficient alternative to the sometimes expensive evaluation of anatomical or functional parameters of the organ of interest.

## 1. Introduction

In the last decades, the development of computational models able to account for personalized data has proven to be an essential tool in the path to precision cardiology (Lamata et al., 2014). When applied to large cohorts of patients, these models allow to perform *in-silico* clinical trials on the so-called digital twins (Lopez-Perez et al., 2019; Corral-Acero et al., 2020; Gillette et al., 2021; Peirlinck et al., 2021), which can focus on target sub-populations (Lange et al., 2016) for particular applications. One enabling pillar to *in-silico* analysis is the availiability of 3D image datasets, acquired via techniques such as Computerized Tomography or Magnetic Resonance Imaging scans. These techniques provide a spatial description from which the organs of interest are segmented and, typically, transformed into a mesh which can be used to provide patient-specific or population representative computer models (Lopez-Perez et al., 2015).

However, clinical adoption of digital twin technologies is limited to the scarce availability of clinical anatomical data with enough level of detail, specially in the case of rare conditions. The segmentation of clinical images is time-consuming and suffers from observer variability. Despite the promising results in the automation of the process via machine learning approaches (Bratt et al., 2019; Hepp et al., 2020; Gilbert et al., 2021), these models fail to generalize well if the object of interest is infrequent in the population. Thus, in clinical practice, image processing and segmentation remains mostly a semi-automatic task. Finally, common approaches used to build virtual populations are based on statistical shape modeling (Young and Frangi, 2009; Casciaro et al., 2014), or other descriptive statistics that rely on the geometrical variability of the samples, that is, are *data-driven* (Rodero et al., 2021). As opposed, *clinically-driven* approaches must produce virtual cohorts with a common underlying clinical characteristic or phenotype, and typically depend on the anatomical or functional properties of the organ of interest (Romero et al., 2009; Britton et al., 2013).

Some studies based on statistical shape modeling, i.e., data-driven approaches, have tried to find correlations with anatomical phenotype. In Cosentino et al. (2020), they aimed to explore the aortic morphology and the associations between shape and function, obtaining shape modes that were associated to specific morphological features of aneurysmal aortas. In Bruse et al. (2017), unsupervised hierarchical clustering was used to cluster anatomical shape data of patient populations to obtain clinically meaningful shape clusters of aortic arches. More recently, Thamsen et al. (2021) developed a clinically-oriented methodology for generating a large database of synthetic cases to train machine learning models, with characteristics similar to clinical cohorts of patients with coarctation of the aorta. In that case, in addition to the geometrical data, flow fields and simulation results were used to define the virtual cohorts, by filtering out the virtual population samples that did not meet some clinical restrictions. This is a common approach, since random generation of individuals does not guarantee that the resulting anatomic case will be physiologically plausible or will belong to the target population. Thus, the generated sample has to be filtered through different *acceptance criteria*, which can range from mere outlier rejection, based on a real observed cohort when available, to more sophisticated tests to restrict the sample to a particular phenotype (Niederer et al., 2020).

Nonetheless, the application of acceptance criteria implies that part of the effort done to generate and assess synthetic cases will be discarded. This waste of effort can be dramatic if the acceptance criteria are computationally demanding, e.g., if the decision depends on the result of a Computational Fluid Dynamics simulation (Thamsen et al., 2021), or if the target cohort is infrequent in the population.

The main goal of this paper is to assess different strategies to increase the *efficiency* of the generation of thoracic aorta cohorts, understanding the efficiency as the ratio of accepted cases with respect to the total number of cases generated and evaluated. We focus on *clinically-driven* criteria, and rely on machine learning techniques to accelerate the acceptance criteria evaluation when computationally demanding tasks are involved. The problem can then be recasted into one of classification, where we want to find the anatomies that meet a given criteria before evaluating it.

Considering the cases in which the evaluation of the acceptance criterion is expensive, e.g., when simulations are involved, we propose the substitution of this computation by machine learning surrogates. In particular, we build functions, based on Support Vector Machines (SVM), that predict the outcome of the biomarkers and of the acceptance criteria without having to compute them explicitly. This strategy can substantially accelerate the process in those cases in which the evaluation of the acceptance criterion is computationally demanding.

## 2. Materials and Methods

### 2.1. Problem Statement

In order to properly define the problem we consider a starting cohort, *C*_0_, which is determined by a set of *n*-dimensional vectors, {_a_*i*_}*i* = 1, …, *K*_0__, in some feature space. Each vector ${\text{a}}_{i}\in {\mathbb{R}}^{n}$ represents the codification of the aorta anatomy of an individual. This cohort is a sample of an underlying population $P_{0}$, which corresponds to the set of physiologically viable aortas of the phenotype of interest. The goal of the cohort synthesis problem is to generate a new cohort *C*_1_ = {_a_*j*_}*j* = 1, …, *K*_1__, with *K*_1_≫*K*_0_, and with the property that $C_{1}\subset P_{0}$. In order to decide whether a particular aorta belongs to the population, we can use whatever prior information we have about it, which can range from the statistical plausibility of a particular vector, compared to the original cohort, to the evaluation of its anatomical or functional properties. We can express this by means of a acceptance function, $A:{\mathbb{R}}^{n}\to \left\{0,1\right\}$, with $A\left(\text{a}\right)=1\Leftrightarrow \text{a}\in P_{0}$. Provided that we have some computable estimation of A, and following the scheme depicted by Niederer et al. (2020), the procedure can be barely described as: draw vectors a_*j*_, and add them to *C*_1_ if A(a)=1, until |*C*_1_| = *K*_1_.

**Figure 1:**
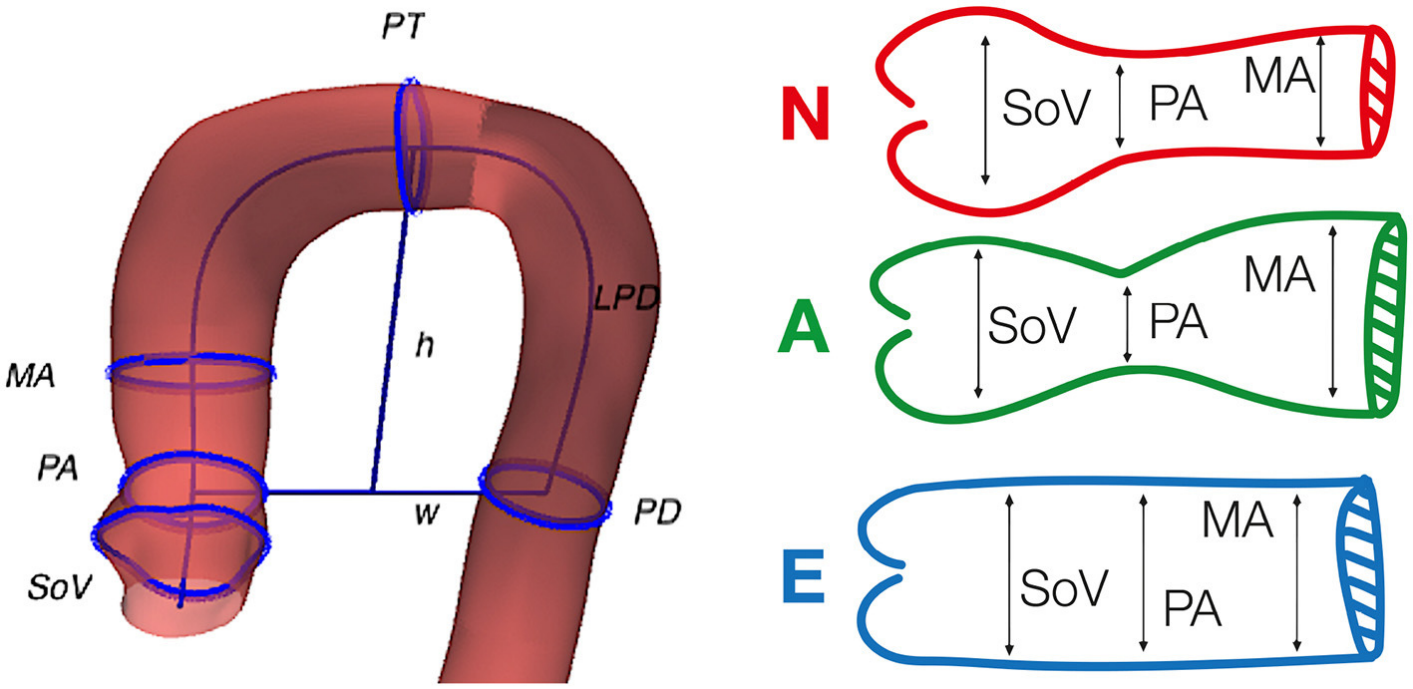
Geometric biomarkers and phenotypes used in the study. Left, graphical representation of some of the considered anatomical biomarkers superimposed on the anatomy of an aorta. Right, the three phenotypes defined by Schaefer et al. (2008), that are used in the clinically-driven cohort generation. The reader can refer to Table 1 for the detailed meaning of the acronyms.

**Figure 2:**
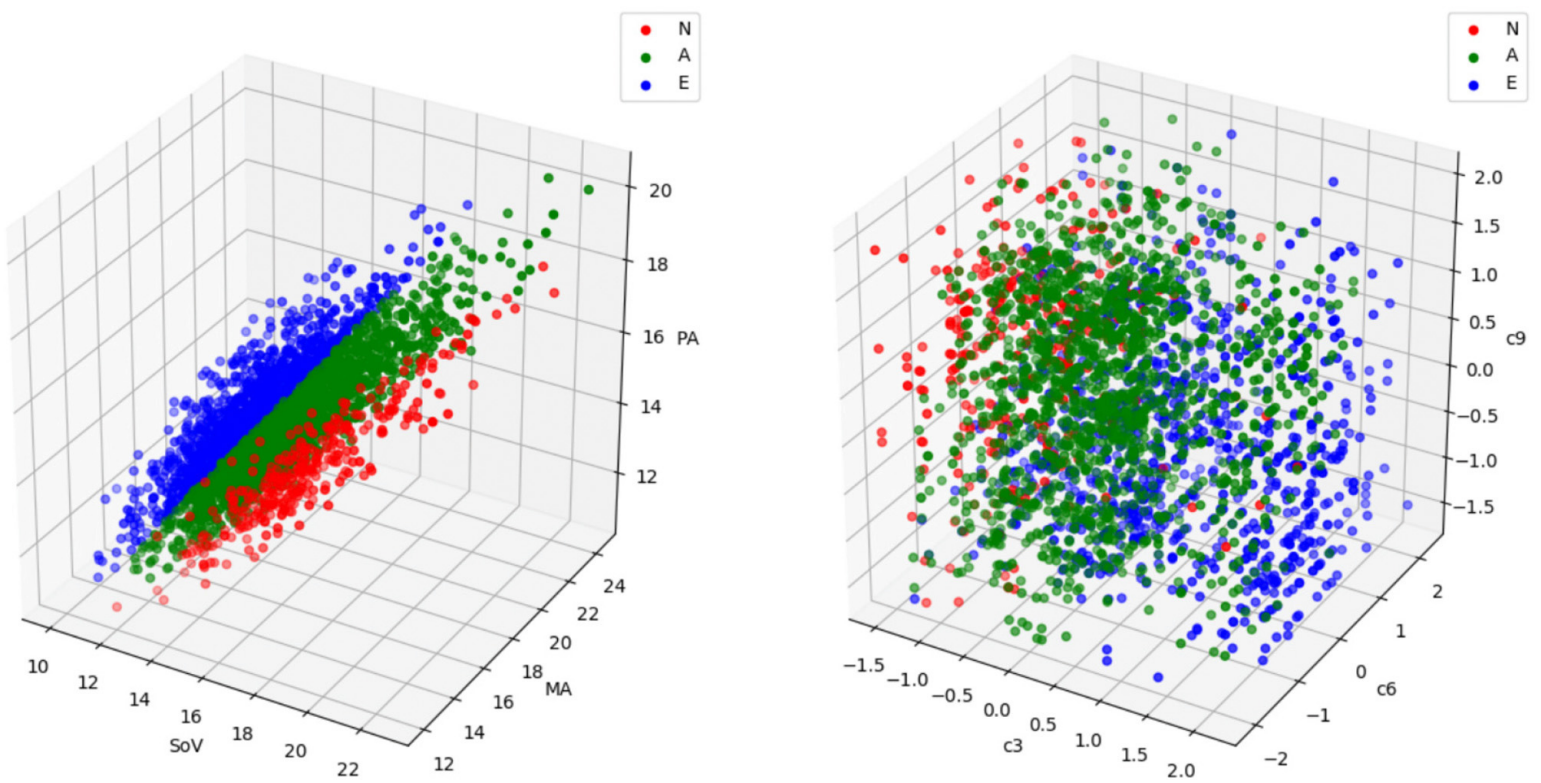
A sample with the three aortic root phenotypes (labeled as *N*, *A*, and *E*) defined in Schaefer et al. (2008) represented in the biomarkers space (left) and in the feature space (right). Each point represents an aorta. In the biomarkers representation, the coordinates correspond to the three biomarkers involved in the phenotype definition, in millimeters (refer to Figure 1 and Table 1 for acronym meanings and phenotype definitions). In the feature space representation, the coordinates are the coefficients of the three deformation modes, *c*_3_, *c*_6_ and *c*_9_, that are most discriminant in this problem. Phenotype *N* is represented in red, phenotype *A* in green and phenotype *E* in blue. While in the biomarkers space the three phenotypes are clearly separable, the region occupied by a particular group in the feature space is much harder to identify and exploit for cohort synthesis.

As one can expect, this problem has a small efficiency ratio using a simple draw-and-test strategy. As an example, let us consider the problem of generating a cohort of patients from one of the three disjoint phenotypes proposed by Schaefer et al. (2008). In that study, the authors classify the aortic root based on the relationship between the radius of the Sinuses of Valsalva, the sino-tubular junction and the mid-ascending aorta (Figure 1, left, shows the definition of the three phenotypes). Given an initial cohort *C*_0_ containing the three phenotypes in different proportions, the goal of the experiment is to generate a virtual cohort that only includes one of them. Figure 2 shows that, even though aortas of the three classes can be easily separated in the clinical biomarkers space (Figure 2, left), the distribution of a particular class in the feature space can be much harder to infer. If the target class has a low relative frequency (e.g., phenotype *N* has a frequency below 15%), then a simple draw-and-accept strategy will lead to a very low success ratio. Our study addresses this limitation by reviewing several sampling methods and assessing them in terms of efficiency. In addition, we propose the use of machine learning surrogates to reduce the number of acceptance function evaluations.

### 2.2. Geometric Aorta Representation

For this study, we used a retrospective dataset of 26 thoracic aortas that corresponded to patients with ascending aorta aneurysm. The patients, with ages ranging from 78 to 89 years old, were diagnosed with aortic valve stenosis and were prescribed a valve implantation. Data had been previously segmented manually by expert radiologists from the Computerized Tomography scans in the mesosystole phase of the cardiac cycle prior to the intervention. The supra-aortic branches were removed in all the cases. The final input data used in this study was the set of 26 anonymized triangular surface meshes. This original dataset from which meshes were segmented met the requirements of the Declaration of Helsinki and was approved by the institutional ethics committee.

In our study, we represent the aorta geometry following the approach described by Romero et al. (2019), which is partly equivalent to the description proposed by Meister et al. (2020). The representation starts by approximating the centerline of the thoracic aorta as a cubic B-spline, α:[0, 1] → ℝ^3^. Then, for each point on the wall x, we compute the closest point on the centerline, α(*s*), and its polar coordinates, (θ, ρ), in a local reference frame, < t, v_1_, v_2_> centered in α(*s*), with t the unitary tangent to the curve. After building a set of points (*s*, θ, ρ), we compute a bivariate cubic polynomial that fits ρ as a function of (*s*, θ). Using this information, any point on the surface can be parameterized as

(1) $$\begin{array}{r} \text{x}\left(s,\theta \right)=\alpha \left(s\right)+\rho \left(s,\theta \right)\left(\cos\left(\theta \right){\text{v}}_{1}\left(s\right)+\sin\left(\theta \right){\text{v}}_{2}\left(s\right)\right),\\ s\in \left[0,1\right],\theta \in \left[0,2\pi \right]. \end{array}$$

A high dimensional feature vector for the aorta anatomy is formed by the coefficients of the polynomials that approximate the centerline and the radius in Equation (1). Based on this representation, any anatomy in a cohort of aortas can be described using a mean aorta plus the sum of a reduced set of deformation modes, computed with a Principal Component Analysis (PCA). Each mode of deformation is a high dimensional feature vector that, when added to the mean aorta feature vector, leads to a variation of shape that is relevant in the observed cohort. The representation of a specific aorta in feature space consists of the set of coefficients corresponding to the modes of deformation [the reader can refer to the work by Varela et al. (2017) for further detail on the approach]. In our experiments, we will use low dimensional feature vectors obtained in this feature space generated by the PCA. The dimensionality will be chosen so that it is able to explain a substantial part of the observed shape variation and to account for the particular anatomical traits that are relevant in the experiments. In all the experiments, the feature space that will be used is the one identified from the cohort or real patients' anatomies. This approach is often referred to as Statistical Shape Modeling (Cootes et al., 1995), where the set of shapes that can be described by the feature space is limited to the deformation modes observed in the real cohort. Thus, there is no guarantee that there is a feature vector accurately representing a given anatomy, specially if its phenotype is very different to those observed.

### 2.3. Anatomical Biomarkers on the Aorta

In order to define different acceptance criteria and target phenotypes for which to generate synthetic cohorts, we are going to use a set of 11 anatomical biomarkers of the thoracic aorta, previously described in the literature (Schaefer et al., 2008; Craiem et al., 2012; Casciaro et al., 2014; Bruse et al., 2016; Liang et al., 2017; Sophocleous et al., 2018). Table 1 gives a description of the biomarkers used here, while Figure 1 shows some of them sketched over the anatomy of an aorta from the original cohort. For each feature vector, the set of 11 biomarkers are computed automatically after reconstructing its geometry. Those biomarkers that are defined as the radius of a cross-section are always computed as the semi-major axis of the best fitting ellipse.

**Table 1:** List of biomarkers used to describe the thoracic aorta geometry.

**Label**	**Biomarker description**
SoV	Radius of the aorta in the middle of the sinuses of Valsalva (*mm*)
PA	Radius at a point in the ascending aorta, close to the sinotubular junction (*mm*)
MA	Radius at a point in mid ascending (*mm*)
PT	Radius at a point in the top of the aortic arch (*mm*)
PD	Radius at a point in the descending aorta, opposite to PA (*mm*)
LPD	Length of centerline from valve to PD (*mm*)
*k*	Mean analytic curvature of the centerline from PA to PD ($\frac{1}{mm}$)
*h*	Height from PT to the level of PA/PD (*mm*)
*w*	Width of the arch, measured as the distance from PA to PD (*mm*)
*h*/*w*	Height-to-width ratio
*tor*	Tortuosity, defined as $1-\frac{W}{LPD}$

### 2.4. Sampling Methodology

The feature space generated after the PCA (described in section 2.2) can also be exploited to draw new random individuals by means of Statistical Shape Modeling (Heimann and Meinzer, 2009). Given a feature vector a = (*a*_1_, …, *a*_*n*_), each component *a*_*i*_ is interpreted as the coefficient associated to the *i*-th deformation mode, and the corresponding anatomy can be reconstructed by adding all these deformation modes to the mean aorta shape. Thus, by means of the generation of random feature vectors, new anatomies can be synthesized (Liang et al., 2017; Rodero et al., 2021; Thamsen et al., 2021). We have grouped the sampling strategies in three main categories: non-parametric sampling, parametric sampling and Neural Network based generation.

#### 2.4.1. Non-parametric Sampling

If we have a small sample, we can make use of a bootstrapping technique. Bootstrapping allows to generate a new sample of larger size, with similar statistical properties to the original reduced dataset (Efron, 1979; Efron and Tibshirani, 1993). Essentially, bootstrapping generates a new feature vector a = (*a*_1_, …, *a*_*n*_) in which each component is chosen randomly from the observed values in the original, small sample. Starting from the reference cohort of size *K*_0_ = 26, formed by aortas of real patients, we project their geometric description onto the reduced dimension feature vector. Next, using the coordinates of the resulting *K*_0_ feature vectors we generate a larger size cohort using bootstrapping.

#### 2.4.2. Parametric Sampling

An alternative to non-parametric sampling is to assume some hypothesis on the probability distribution of each coefficient, *a*_*i*_. Then, the hyper-parameter of the distributions can be inferred from the original sample. Synthetic samples can be directly drawn with pseudo-random number generator that mimics the inferred distribution. In this work we used multivariate Gaussian distribution, which is typically assumed when dealing with natural phenomena, and uniform distribution typically related to Monte Carlo experiments. Given the multidimensional dataset, ${\left\{\text{a}\right\}}_{i=1}^{K_{0}}$, where ${\text{a}}^{i}=\left(a_{1}^{i},\ldots ,a_{n}^{i}\right)$, the Gaussian distribution, N(μ,Σ) is determined by the mean, μ and covariance matrix Σ, that can be estimated with the sample mean $\bar{\text{a}}=\frac{1}{K_{0}}\overset{K_{0}}{\underset{i=1}{\sum }}{\text{a}}^{i}$ and the sample covariance matrix $\text{Q}=\frac{1}{K_{0}-1}\overset{K_{0}}{\underset{i=1}{\sum }}\left({\text{a}}^{i}-\bar{\text{a}}\right)\cdot {\left({\text{a}}^{i}-\bar{\text{a}}\right)}^{T}$. The uniform distribution, $U\left(l_{j},u_{j}\right)$ is determined by the lower and upper extrema of the intervals for each dimension 1 ≤ *j* ≤ *n*. Sample min, $l_{j}=\underset{1\leq i\leq K_{0}}{\min}\left\{a_{j}\right\}$ and max,$u_{j}=\underset{1\leq i\leq K_{0}}{\max}\left\{a_{j}\right\}$ are used as estimates.

#### 2.4.3. Generative Adversarial Networks

Generative adversarial networks (GAN), proposed by Goodfellow et al. (2020), offer a sample generation strategy from the machine learning perspective. The model consists of a combination of two artificial neural networks, a Generator (G) and a Discriminator (D). The generator uses a density distribution to generate new data. Opposed to the generator, the discriminator acts as a classifier trying to detect whether the observed data are coming from G or come from the real dataset. This learning model is based on the zero-sum or minimax strategy for non-cooperative games. Within this learning strategy, D is trying to maximize its accuracy at classifying data between real and fake, while, G is trying to minimize D's accuracy, by fooling it. A GAN model converges when discriminator and generator reach a Nash equilibrium, or optimal point for the minimax problem. For the use of GAN to generate new samples we start with the reference cohort *C*_0_ for which we want to obtain a larger sample. During the training process, we will present to the GAN the observed feature vectors as real samples.

### 2.5. Cohort Generation Experiments

Each experiment will be aimed to evaluate the efficiency of the different sampling methodologies in a particular scenario. Each scenario is defined by a reference cohort *C*_0_ (with size *K*_0_) and an acceptance function $A:{\mathbb{R}}^{n}\to \left\{0,1\right\}$ that takes the value 1 on a feature vector a if and only if a represents an aorta that meets the defined acceptance criterion. During the experiment, a sample *C*_1_ of size *K*_1_≫*K*_0_ aortas will be generated and evaluated using A. The outcome of the experiment for a given sampling strategy will be an efficiency ratio defined as

(2) $$\begin{array}{r} e=\frac{\left|\left\{\text{a}\in C_{1}:A\left(\text{a}\right)=1\right\}\right|}{K_{1}}. \end{array}$$

**Figure 3:**
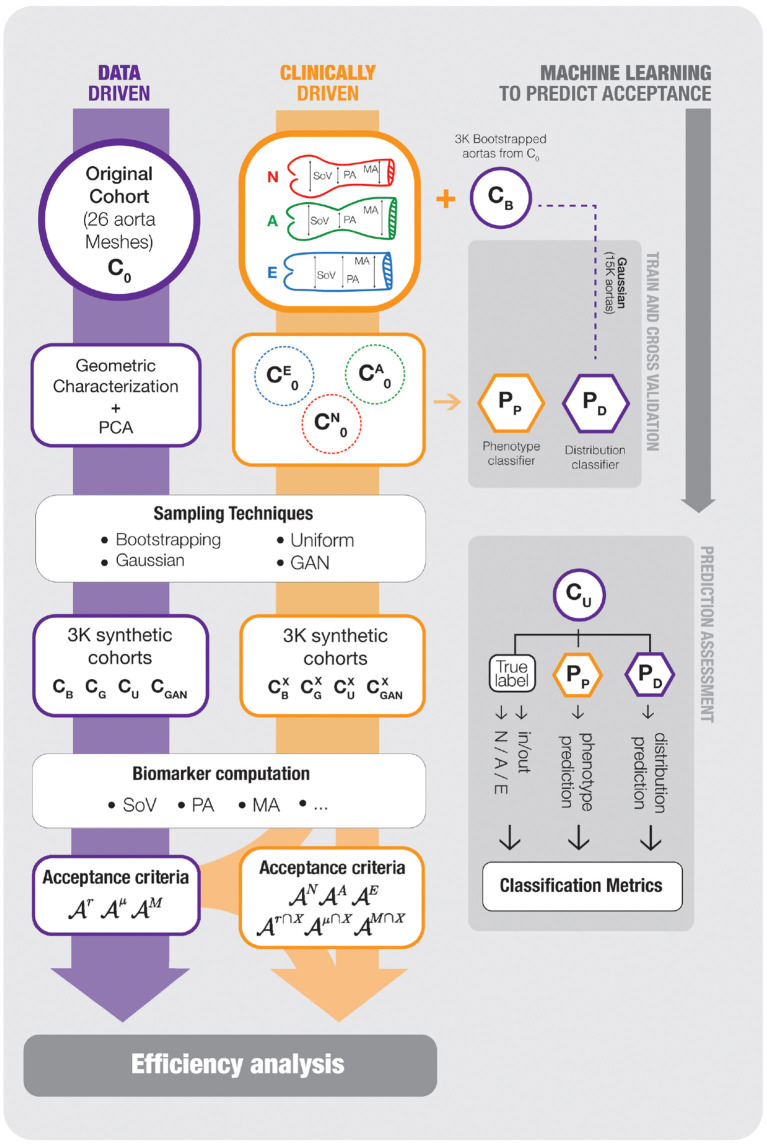
An scheme of the workflow followed in our experimental setup. On the left track, the data-driven cohort generation scenario is shown; the reference cohort *C*_0_, that is characterized using PCA to generate new samples *C*_*B*_, *C*_*G*_, *C*_*U*_, and *C*_*GAN*_, which are assessed with the acceptance functions $A^{X}$. The middle track, representing the clinically-driven experiments, starts splitting the samples of the boostrapping cohort, *C*_*B*_, generated on the previous scenario, onto the three target phenotypes, *N*, *A* and *E* and, then, new cohorts $C_{B}^{X}$, $C_{G}^{X}$, $C_{U}^{X}$ and $C_{GAN}^{X}$ are generated and, again, assessed by the corresponding acceptance functions $A^{X}$. Finally the rightmost blocks represents the development of Machine Learning surrogates to predict the acceptance functions. The synthetic cohort *C*_*B*_ is used to train two SVM models, *P*_*p*_ and $P_{d}^{\mu }$ (*P*_*D*_ in the chart), that predict the outcome of $A^{\mu }$ and the aorta phenotype, respectively. The models are evaluated with *C*_*U*_, that was not used during training. For any item of the picture, a purple frame means data-driven and an orange frame means clinically-driven. The reader can refer to the text for further detail.

Based on this common scheme we define three main scenarios. Data-driven cohort generation, clinically-driven cohort generation and feature space acceptance criterion usage. Next, we describe these three problems, together with the different acceptance criteria that are used. Each experimental setup is repeated for all the sampling strategies discussed in section 2.4. The acceptance functions involve the set of biomarkers described in section 2.3. Thus, the evaluation of an acceptance function involves the reconstruction of the surface of the aorta, from the description defined in section 2.2, and the automatic computation of the biomarkers. Figure 3 shows an scheme of the workflow along with the complete set of experiments that are performed.

#### Problem 1: Data-Driven Acceptance Criteria

Given a reference cohort *C*_0_, we consider *data-driven* cohort synthesis as the sampling of a larger cohort *C*_1_ with the only acceptance criteria of being compatible with *C*_0_. In this first set of experiments the acceptance function must be some measure of how likely is a particular observation a, provided that it belongs to the same population from which *C*_0_ was drawn. In our data-driven cohort generation experiments, the reference cohort is the sample formed by 26 aorta geometries acquired from real patients described in section 2.2. The generated cohort will have a size *K*_1_ = 3, 000.

The particular definition of A can depend on our goals when generating *C*_1_; e.g., if we want to simulate and assess the effect on a biomarker of a clinical intervention, we will favor a cohort that provides a good statistical description of the underlying population; on the contrary, if *C*_1_ is to be used as the training set for a nonlinear model, then we may require that the population is more evenly sampled to prevent unbalanced classes, regardless the actual frequency of each group in the population. For this reason we use three acceptance functions and present the results for discussion, indicating in which contexts they could be of interest. All three criteria are based on an acceptance interval for the values of the biomarkers, and differ in the way this interval is defined.

The first acceptance criterion is based on the mere range of the observed biomarkers in *C*_0_. In order to accept a feature vector a, the associated geometry must have all biomarkers within the observed ranges. We will refer to the acceptance function for this criterion as $A^{r}$.

The second acceptance criterion takes into account the dispersion observed in the original cohort to perform a sort of outlier rejection. More precisely, we define intervals that accumulate the 95% of the probability of finding each biomarker. In absence of any other information about the actual distribution of the different biomarkers, we rely on Chebyshev's theorem. This theorem sets a bound for the probability accumulated in the tails of a distribution based on the mean μ, the variance σ and the mode *M*. Assuming unimodality, Chebyshev's theorem establishes that the interval defined by *M*±3*B*, where $B=\sqrt{\sigma^{2}+{\left(M-\sigma \right)}^{2}}$ contains at least 95% of the area under the probability function^1^. Refer to the work by Amidan et al. (2005) for further detail. The acceptance criterion is met by a vector if all the biomarkers fall into the corresponding interval, computed with the estimators on the sample *C*_0_. We will refer to the associated acceptance function as $A^{M}$.

The third acceptance criterion assumes normality in the distribution of the biomarkers. Since we are dealing with a sample of a natural population it is reasonable to consider the possibility that at least some of the biomarkers follow a Gaussian distribution. Since the previous acceptance criterion is rather permissive, as it is completely agnostic of the probability density functions of the biomarkers, we consider pertinent adding a more restrictive acceptance interval. Thus, we add the criterion that all the biomarkers of a synthetic aorta must lie within two standard deviations from the mean, and denote the corresponding acceptance function $A^{\mu }$.

Table 2 presents the values of the statistics for the different biomarkers proposed and the resulting intervals defined by the three acceptance criteria. These data correspond to the sample statistics of the observed cohort *C*_0_ formed by the 26 patient derived anatomies.

**Table 2:** Statistical description of each one of the biomarkers measured on the aorta.

**Biomarker**	**μ**	***M***	**σ**	***B***	**[min, max] ($A^{r}$)**	***M±3B ($A^{M}$)***	**μ±2σ ($A^{\mu }$)**
PA	14.77	14.71	1.40	1.40	[12.38,17.50]	[10.50,18.92]	[11.97,17.57]
PD	11.96	11.35	1.12	1.27	[10.24,14.32]	[7.53,15.17]	[ 9.73,14.20]
PT	13.38	12.78	1.55	1.67	[10.19,17.32]	[7.78,17.78]	[10.28,16.49]
LPD	233.69	211.36	25.25	33.71	[175.4,274.16]	[110.22,312.49]	[183.19,284.20]
MA	17.28	15.19	1.79	2.75	[15.19,21.5]	[6.95,23.44]	[13.71,20.85]
SoV	15.30	15.49	2.12	2.13	[11.78,21.0]	[9.10,21.89]	[11.06,19.54]
k	7.42	7.49	0.44	0.45	[6.74,8.40]	[6.15,8.84]	[6.53,8.30]
h	92.12	82.80	15.21	17.85	[70.21,139.42]	[29.26,136.33]	[61.69,122.55]
w	70.72	60.70	10.76	14.71	[50.25,88.56]	[16.56,104.83]	[49.19,92.25]
h/w	1.33	1.18	0.29	0.32	[0.91,1.91]	[0.22,2.15]	[0.76,1.90]
tor	0.70	0.71	0.02	0.02	[0.66,0.73]	[0.63,0.78]	[0.65,0.74]

*From left to right, the first four columns correspond to the mean, μ, the mode M, the standard deviation, and the 95% Chebyshev's theorem bound. The last three columns contain the intervals considered in the different acceptance functions $A^{r}$, $A^{M}$, and $A^{\mu }$ (further detail can be found in the text). All units are in millimeters, except for k, which is expressed in mm^−1^, and h/w and tor which are adimensional*.

As we stated earlier, in some contexts it is important that the distribution of a biomarker in the virtual cohort is a good estimation of the original population distribution, e.g., when we want to draw conclusions about the probability of certain output variable that results from that biomarker. To provide some insight on this regard, we will perform an additional test on the generated cohorts. In order to detect if the distribution of the biomarkers differ from that in the original sample, we carry out a Mann-Whitney-Wilcoxson's hypothesis contrast test (MWW) on the observed distribution of each one of them. Note that this test will not be involved on the computation of the efficiency of the sampling methods, but will point toward their possible loss of statistical fidelity.

#### Problem 2: Clinically-Driven Acceptance Criteria

We refer to clinically-driven cohort generation as the process of generating a sample *C*_1_ with an acceptance criterion that is not based on a reference cohort *C*_0_, but on a clinical requirement. This does not mean the absence of *C*_0_, but only that the acceptance function will not depend on the statistical properties of *C*_0_. If a reference cohort is used, e.g., to estimate the parameters for parametric sampling, then it has to be taken into account that it can bias the generation process. We are interested in the case in which we do not have access to a representative sample of the target population. In this case, we can use a sample from a larger population that contains the subpopulation defined by the acceptance criterion.

In order to set several acceptance criteria for our experiments, we refer to the phenotype classification of the aortic root defined by Schaefer et al. (2008). In their work, they consider three disjoint classes according to the radius of the sinuses of Valsalva (SoV), of the sino-tubular junction (PA) and of a point in mid-ascending aorta (MA). The three phenotypes are defined as:

- Phenotype *N* : SoV > PA and SoV >= MA,
- Phenotype *A* : SoV > PA and SoV < MA,
- Phenotype *E* : SoV < = PA.

In our experiments we will use a different reference cohort for each phenotype. Since the observed sample of real aortas is too small to have a proper representation of the three phenotypes, we will rely on a bootstrapped sample of size *K*_0_ = 3, 000 obtained by resampling the clinical cohort. Let *C*_*B*_ be this sample. Given a phenotype *X*∈{*N, A, E*}, we define its reference cohort as $C_{0}^{X}=\left\{\text{a}\in C_{B}:\text{a}\text{is of phenotype}X\right\}$.

We will evaluate the efficiency of the sampling strategies studied by generating a new sample of size *K*_1_ = 1, 000 for each phenotype and augmentation method. Then, we analyze the results obtained from three different points of view, clinically-driven criteria, data-driven criteria, and the intersection of both. We do this for phenotype *X*∈{*N, A, E*} as follows; first, the phenotype acceptance criterion $A^{X}$, that accepts an aorta if it belongs to phenotype *X*, will be checked; also, the three acceptance criteria defined for the data-driven cohort will be measured ($A^{r}$, $A^{\mu }$ and $A^{M}$); and, finally, simultaneously data-driven and clinically-driven criteria are evaluated, retaining aortas that meet both, $A^{r\cap X}$, $A^{\mu \cap X}$, and $A^{M\cap X}$.

#### Problem 3: Feature Space Acceptance Criteria

If the acceptance ratio is low during the sampling process, generating large cohorts can involve a really high burden. In order to reduce the amount of unsuccessful evaluations of the acceptance criteria, our last proposal is to substitute the acceptance functions A by efficient surrogates that provide a prediction of the outcome of A without actually evaluating them.

In the context of our work, we will train two models to predict the outcome of some of the acceptance functions that have been defined: a function $P_{d}^{\mu }:{\mathbb{R}}^{n}\to \left\{0,1\right\}$ that predicts if a feature vector a∈ℝ^*n*^ will be accepted by $A^{\mu }$, and a function $P_{p}:{\mathbb{R}}^{n}\to \left\{N,A,E\right\}$ that predicts the phenotype of the aorta associated to a feature vector a∈ℝ^*n*^. $P_{d}^{\mu }$ will be based on a Support Vector Machine (SVM), while *P*_*p*_ will be a Support Vector Classifier (SVC) that, internally, uses several one-vs.-one SVM classifiers to decide the class (Bishop, 2006). After the predictors have been trained, they can be used to evaluate very efficiently every feature vector that is drawn during the sampling process. Then, only those anatomies that have passed the first evaluation are then assessed by the real acceptance function. Note that, in the previous two experiments, the efficiency was measured in terms of the amount of feature vectors generated. Now, when the evaluation of A is substantially higher than the random generation of a vector, the efficiency can be defined as the ratio between the number of successful evaluations of A and the total number of evaluations of A. As a consequence, the efficiency of the overall process will be the *sensitivity* or *recall* of the SVM predictor.

To test this approach, the two predictors will be trained using cross validation over generated cohorts of aortas where no acceptance criterion has been applied. In addition, a completely new generated cohort will be used as a final test set. The rightmost part of Figure 3 shows the populations and schemes used to train and test the two different classifiers proposed in this section.

## 3. Results

**Figure 4:**
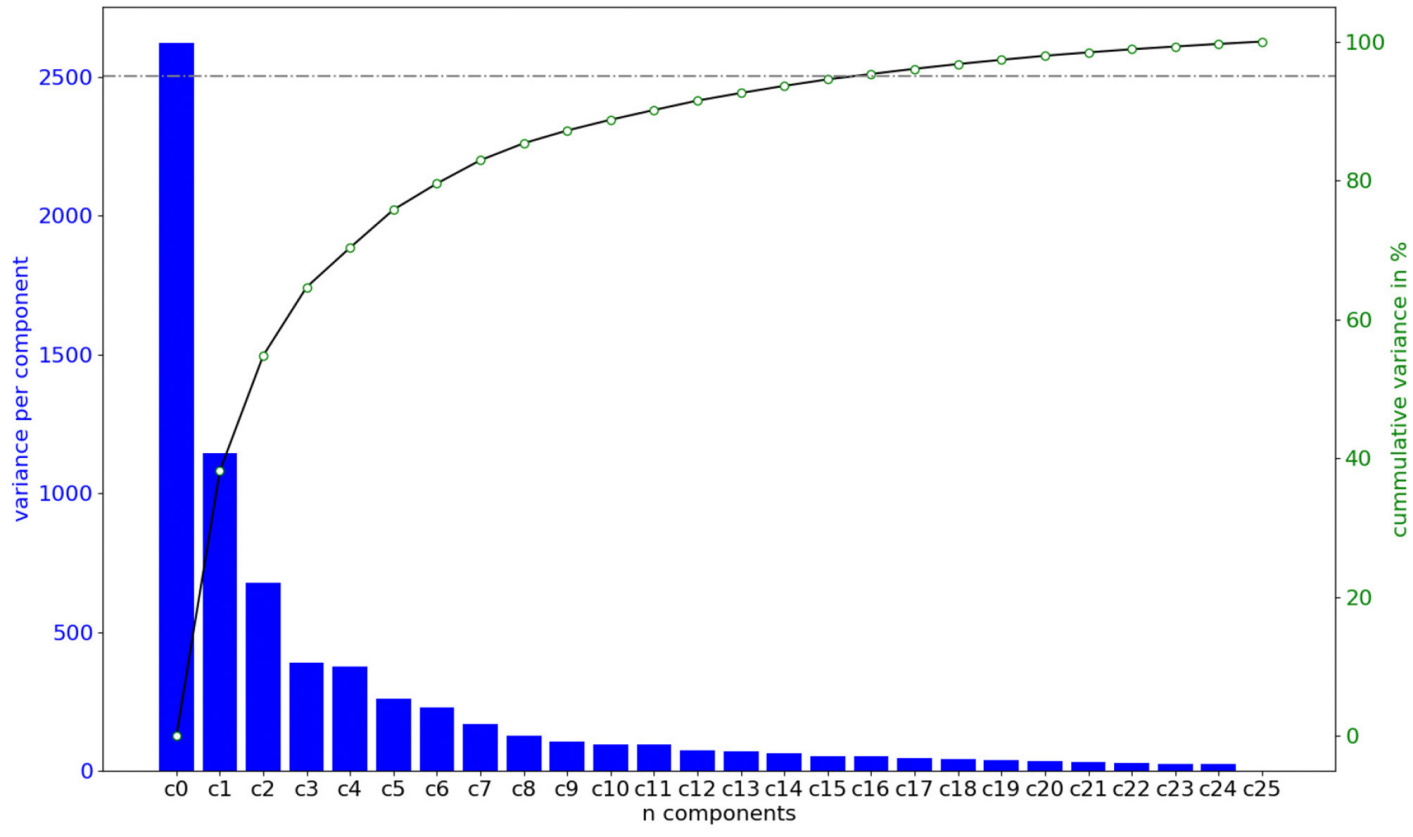
Amount of shape variation explained by each of the components of the feature vector in the space defined by the PCA, in order of importance. Importance is computed by training a Random Forest model and computing the decrease of impurity of each subtree. The first *n* = 16 features are capable of explaining 95% of the shape variation.

Figure 4 shows the variance associated to each mode of deformation, and the accumulated variation explained by considering the first *n* features of the PCA. The first 16 variation modes can explain 95% of the anatomical variability in the observed sample of 26 aortas. Moreover, a correlation analysis indicates that the R^2^ between the 16 first PCA modes and the 3 biomarkers of interest that define the clinical acceptance criteria PA, MA, and SoV, are 93.4, 97.4, and 96.9%, respectively, indicating that the 16 dimensional space is an adequate basis to tackle the problem.

### 3.1. Data-Driven Acceptance Criteria

After performing the PCA on the reference cohort *C*_0_, of size *K*_0_ = 26, we have generated a cohort *C*_1_ of *K*_1_ = 3, 000 synthetic shapes by sampling the space using several methods: bootstraping, uniform sampling, Gaussian sampling and a GAN. These cohorts are represented in Figure 3 as *C*_*B*_, *C*_*U*_, *C*_*G*_ and *C*_*GAN*_, respectively. In the case of the GAN, it was trained increasing the epochs from 100 until it reached stability in the accuracy, which was met with a total of 2,000 epochs. The size of the batches used in each epoch was set to 5, that is approximately a fifth of the size of the cohort *C*_0_.

**Figure 5:**
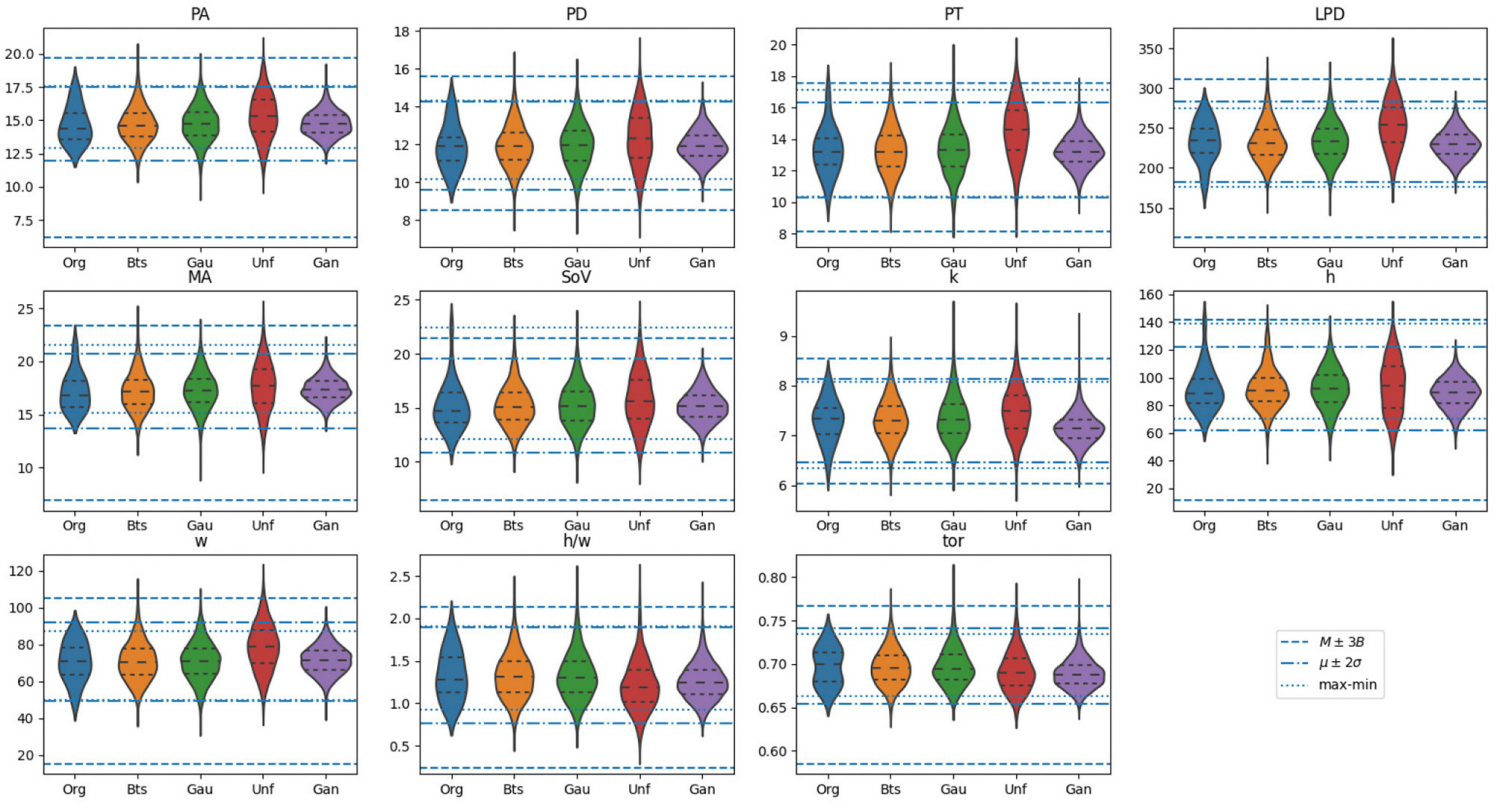
Violin plots for the distribution of biomarkers on the original samples, alongside with those generated using the proposed methods: Bootstrap (Bts), Gaussian (Gau), Uniform (Unf) and generative adversarial network (GAN). Horizontal lines mark the bounds for the different acceptance criteria defined in section 2.5: $A^{r}$, with dotted lines, $A^{\mu }$, with dash-doted lines, and $A^{M}$ with dashed lines. The units of the vertical axis are in millimeters, except for biomarkers *k*, which is expressed in *mm*^−1^, and *h*/*w* and *tor* which are a dimensional.

For all the aortas in the synthetic cohorts, the different biomarkers were computed. The resulting biomarker distributions are presented in Figure 5 by means of violin charts. Horizontal lines in the figure mark the bounds for the different acceptance criteria defined in section 2.5: $A^{r}$, with dotted lines, $A^{\mu }$, with dash-doted lines, and $A^{M}$ with dashed lines. The figure shows that all sampling methods generate distributions of the biomarkers that surpass the range defined by $A^{r}$, higher variability than that observed in the original cohort *C*_0_.

**Figure 6:**
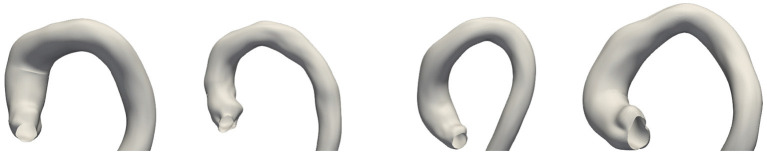
Examples of four synthetic aortas with decreasing feasibility of the biomarkers according to the acceptance functions. From left to right: the first one is accepted by all of the criteria; the second one is rejected by $A^{r}$, but not by the other acceptance functions; the third one is only accepted by $A^{M}$ and the last one is rejected by all the criteria.

One of the properties of the acceptance functions is that, in most cases, $A^{r}$ tends to be the most restrictive one due to the limited variability observed for each biomarker in *C*_0_. Most likely, this is due to the small size of that sample that leaves the tails of the underlying distribution underrepresented. However, there are exceptions, such as in the distribution for SoV or h, where the upper bound is remarkably high compared to that of $A^{M}$ and $A^{\mu }$. Among these two acceptance functions, the criterion based on $A^{\mu }$ is more restrictive than that based on $A^{M}$, which is an expected result based on their definition. Figure 6 shows the anatomy of four synthetic aortas that fall within the different acceptance intervals. From left to right, an aorta that meets $A^{r}$, an aorta that meets $A^{\mu }$ but not $A^{r}$, an aorta that meets $A^{M}$ but not $A^{\mu }$ and an aorta that does not meet any of the criteria.

Table 3 shows the efficiency of each method measured using the different acceptance criteria, as defined in section 2.5. The sampling strategies are arranged in rows, while each column correspond to an acceptance function. In addition, the last column shows the results of applying the MWW hypothesis contrast test to compare the distribution of each biomarker obtained in *C*_1_ to that observed in *C*_0_. Consistently with the ranges observed for $A^{r}$, the acceptance ratio for this criterion is notably smaller than that for the other criteria.

**Table 3:** Results of the generation of synthetic aorta cohorts with different sampling methods.

**Method**	**$A^{r}$**	**$A^{\mu }$**	**$A^{M}$**	**MWW test**
Bootstrapping	0.599	0.786	0.979	0 / 11 reject *H*_0_
Gaussian	0.625	0.739	0.966	0 / 11 reject *H*_0_
Uniform	0.293	0.424	0.893	7 / 11 reject *H*_0_
GAN	0.808	0.950	0.999	2 / 11 reject *H*_0_

*The last column shows the number of biomarker distributions significantly different to the original according to the MWW test (refer to the text for further detail). The columns labeled $A^{\alpha },\alpha \in \left\{r,\mu ,M\right\}$, indicate the efficiency of the data augmentation methods when considering each acceptance function*.

The results show substantial differences between the four sampling strategies, making them suitable for different scenarios. Both Gaussian and bootstrapping sampling show similar efficiency results and are the two that have no biomarker distributions rejected by the MWW test. These distributions would be the most adequate to retain the statistical information of *C*_0_. If we are interested on having a denser representation of any phenotype, despite its actual distribution in the true population, then uniform sampling provides longer tails for the different biomarker distributions. This is at the price of having a very low efficiency if the application of an acceptance function is compulsory; e.g., in the case of considering $A^{\mu }$ more than half the feature vectors are disregarded. If we are considering a clinical scenario in which the biomarkers are the result of costly simulations (Rodero et al., 2021; Thamsen et al., 2021), this has to be taken into account. On the opposite side, the cohort generated by a GAN has the narrowest distributions for all the biomarkers among the three methods, since the generated anatomies are closer to the mean in the PCA space. This results in an efficiency increment on all the criteria, at the price of having much shorter tails and leaving some plausible regions under represented.

### 3.2. Clinically-Driven Acceptance Criteria

We start by building three reference samples, $C_{0}^{X},X\in \left\{N,A,E\right\}$. Our starting point is the bootstrapped synthetic cohort, *C*_*B*_, with size 3, 000, that was generated in the previous section. This sample has been divided in the three reference clinically-based cohorts: $C_{0}^{N}$, of size *K*_0_ = 330 aortas (11% of *C*_*B*_), $C_{0}^{A}$, of size *K*_0_ = 1, 605 aortas (53, 5%), and $C_{0}^{E}$ with the remaining *K*_0_ = 1, 065 aortas (35.5%). For each phenotype, *X*, we take $C_{0}^{X}$ and apply the four sampling methods described in section 2.5 to generate the corresponding synthetic cohort $C_{1}^{X}$ of size *K*_1_ = 1, 000. Even though the definition of the different acceptance functions can be found in section 2.5, for the sake of clarity we present a summary of the meaning of the used criteria in Table 4. Furthermore, we have added subscript *DD* for data-driven criteria and subscript *CD* for clinically-driven criteria.

**Table 4:** List of the acceptance functions that are used in the experiments related to clinically-driven-cohort generation.

**Label**	**Acceptance function description**
$A_{DD}^{r}$	Accepts an aorta if all biomarkers are within the corresponding observed range in *C*_0_
$A_{DD}^{\mu }$	Accepts an aorta if all biomarkers are within the range μ±2σ for that biomarker in *C*_0_
$A_{DD}^{M}$	Accepts an aorta if all biomarkers are within the range *M*±3σ for that biomarker in *C*_0_
$A_{CD}^{X}$	With *X*∈{*N, A, E*}, accepts an aorta if it belongs to phenotype *X*
$A^{\alpha \cap X}$	With α∈{*r*, μ, *M*}, and *X*∈{*N, A, E*}, accepts an aorta if it is accepted by both and $A_{DD}^{\alpha }$ and $A_{CD}^{X}$

*Subscript DD stands for data-driven and subscript CD satnds for clinically-driven. Refer to section 2.5 for a detailed description*.

Results are shown in Table 5. Each row corresponds to a sampling method and a phenotype, and shows the results for that particular synthetic cohort. In the case of the data-driven criteria, the efficiencies have a meaning similar to those in Table 3; it is the ratio of aortas that are plausible according to the observed sample of size *K*_0_ = 26. In the case of the clinically-driven criteria, results can be interpreted like a *confusion matrix* for each method. For instance, in Gaussian sampling and phenotype *N*, a value of 0.141 under $A_{CD}^{A}$ means that 141 of aortas in the synthetic cohort generated to be of class *N*, actually are of phenotype *A*. The *efficiencies* are the elements of the diagonal in each method's block. The last three columns in Table 5 show the result of requiring both a data-driven acceptance function with the acceptance criterion of the phenotype for the row.

**Table 5:** Efficiency values achieved by each method and for each biomarker.

		**Data-driven**			**Clinically-driven**			(**Data** ∩ **Clinically)-driven**		
**Method**	**Phenot**.	**$A_{DD}^{r}$**	**$A_{DD}^{\mu }$**	**$A_{DD}^{M}$**	**$A_{CD}^{N}$**	**$A_{CD}^{A}$**	**$A_{CD}^{E}$**	**$A^{r\cap X}$**	**$A^{\mu \cap X}$**	**$A^{M\cap X}$**
	*N*	0.978	0.650	0.954	0.695	0.301	0.004	0.679	0.457	0.662
	*A*	0.973	0.675	0.957	0.231	0.557	0.212	0.551	0.404	0.529
Bootstrap	*E*	0.969	0.720	0.958	0.008	0.253	0.739	0.724	0.568	0.720

	*N*	0.954	0.753	0.960	0.858	0.141	0.001	0.820	0.655	0.826
	*A*	0.983	0.735	0.968	0.052	0.874	0.074	0.863	0.654	0.845
Gaussian	*E*	0.975	0.722	0.967	0.003	0.117	0.880	0.862	0.646	0.855

	*N*	0.999	0.807	1.000	0.887	0.113	0.000	0.886	0.716	0.887
	*A*	0.979	0.534	0.908	0.135	0.616	0.249	0.605	0.346	0.566
Uniform	*E*	0.954	0.554	0.957	0.009	0.210	0.781	0.753	0.446	0.755

	*N*	0.974	0.806	0.979	0.895	0.102	0.003	0.874	0.736	0.878
	*A*	0.963	0.733	0.958	0.089	0.855	0.056	0.846	0.669	0.833
GAN	*E*	0.931	0.573	0.931	0.000	0.104	0.896	0.840	0.521	0.833

*The efficiency of each row is the result of using the method of the first column in the cohort $C_{0}^{X}$, where X is the phenotype corresponding to the row, as specified in the Phenot. column. Refer to the text for the details on the interpretation of the data. Row colors follow the same convention used in previous figures for phenotypes: red for phenotype N, green for phenotype A, and blue for phenotype E*.

The results indicate that clinically-driven cohort synthesis is a much harder problem than data-driven synthesis, in terms of efficiency. The data-driven columns in the table indicate that, in general, the anatomies generated are within what is observed in *C*_*B*_, even for the uniform distribution. However, the columns for Clinically-driven efficiency point out that a phenotype that is easily identified in the biomarkers space can occupy a region in the feature space that is mangled with aortas of a different phenotype as it was anticipated in section 2.1 and Figure 2.

**Figure 7:**
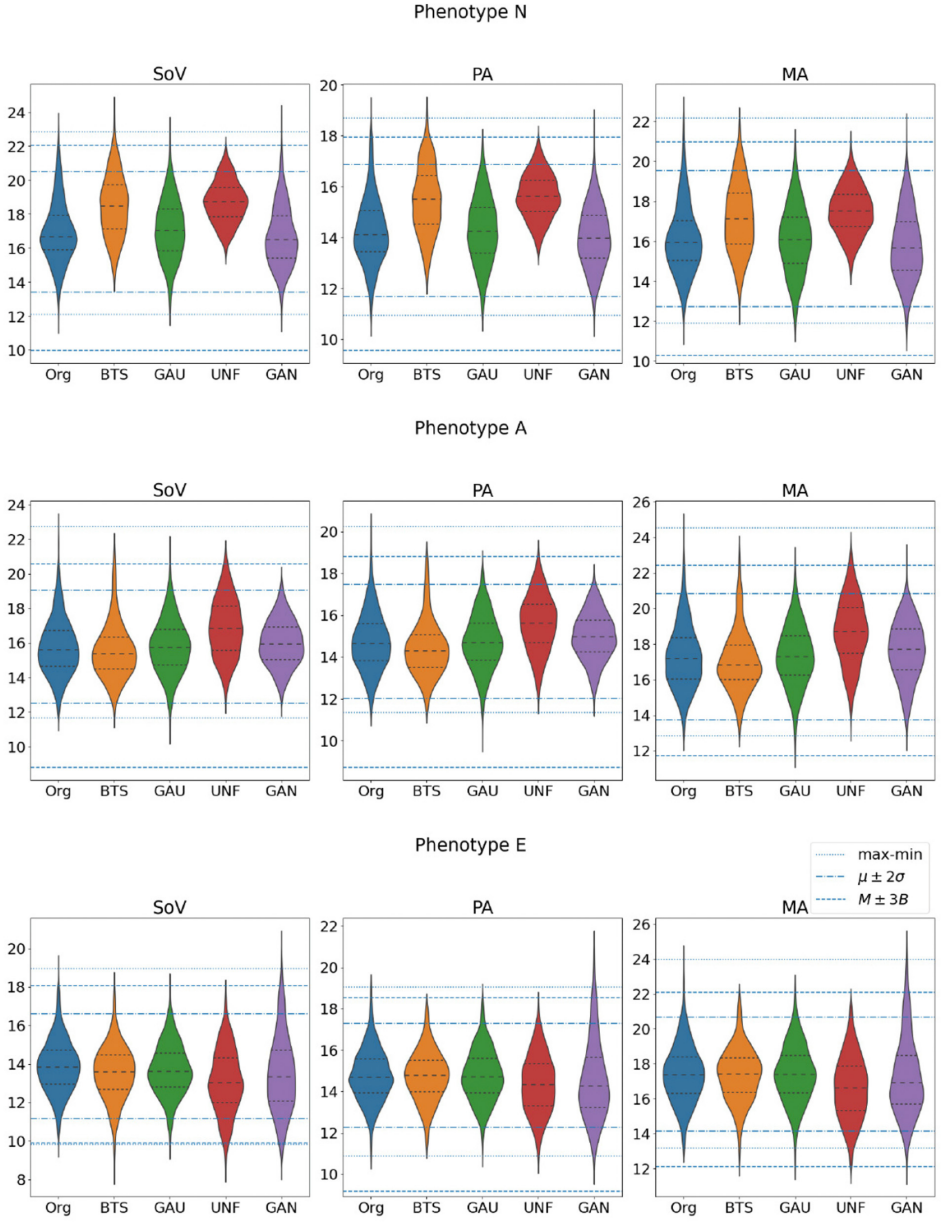
Distributions of the three biomarkers that define the target phenotypes (SoV, PA, and MA) in the set of aortas that actually belong to each one of the three classes. All the values are in millimeters.

Again, the GAN is the sampling strategy that provides a higher efficiency. However, on the contrary to what happened in the data-driven generation, in this case this does not imply narrower biomarker distributions, at least in the three values that define the phenotypes, as it can be inferred from Figure 7. Indeed, this wider span of the distributions leads to lower acceptance ratios in the data-driven criteria.

It also noteworthy that the results can be very dependent on the particular target phenotype in the generated cohort. In our experiments, phenotype *N* is, in general, easier to sample efficiently, while phenotype *A* yields the worst results in all sampling methods except in the Gaussian distribution. This indicates that sampling in the feature space can be very inefficient depending on the target cohort distribution.

### 3.3. Machine Learning Surrogates for Acceptance Criteria

We address now the problem of training predictors for different acceptance functions. The aim of this SVM classifier is to predict if a random sample can be considered as an aorta from the observed distribution of 3,000 bootstrapped aortas. Figure 3 shows the scheme of the training and validation process that is described next.

We start by building a predictor for one of the data-driven acceptance functions; a Support Vector Machine (SVM) model, $P_{d}^{\mu }$ was trained, and acted as a predictor of $A_{DD}^{\mu }$ defined in section 2.5. A large training set of 15, 000 aortas was generated using a Gaussian sampling. The reason for using the Gaussian distribution in this case is that we want a reasonable amount of infeasible aortas in the training set and, according to the results of section 3.1, this is the method that samples best the tails of the biomarkers distributions. To label the elements of this set, the acceptance functions were applied to all of them. In this case, since the original sample of size 26 is small, the statistics used to evaluate $A_{DD}^{\mu }$ have been those obtained from the set *C*_*B*_ of 3,000 bootstrapped aortas. In order to prevent overfitting, a 5 fold cross validation process has been used to train the model. The accuracy obtained in the cross validation process with this model was 0.9 with a radial basis function for the SVM kernel.

**Figure 8:**
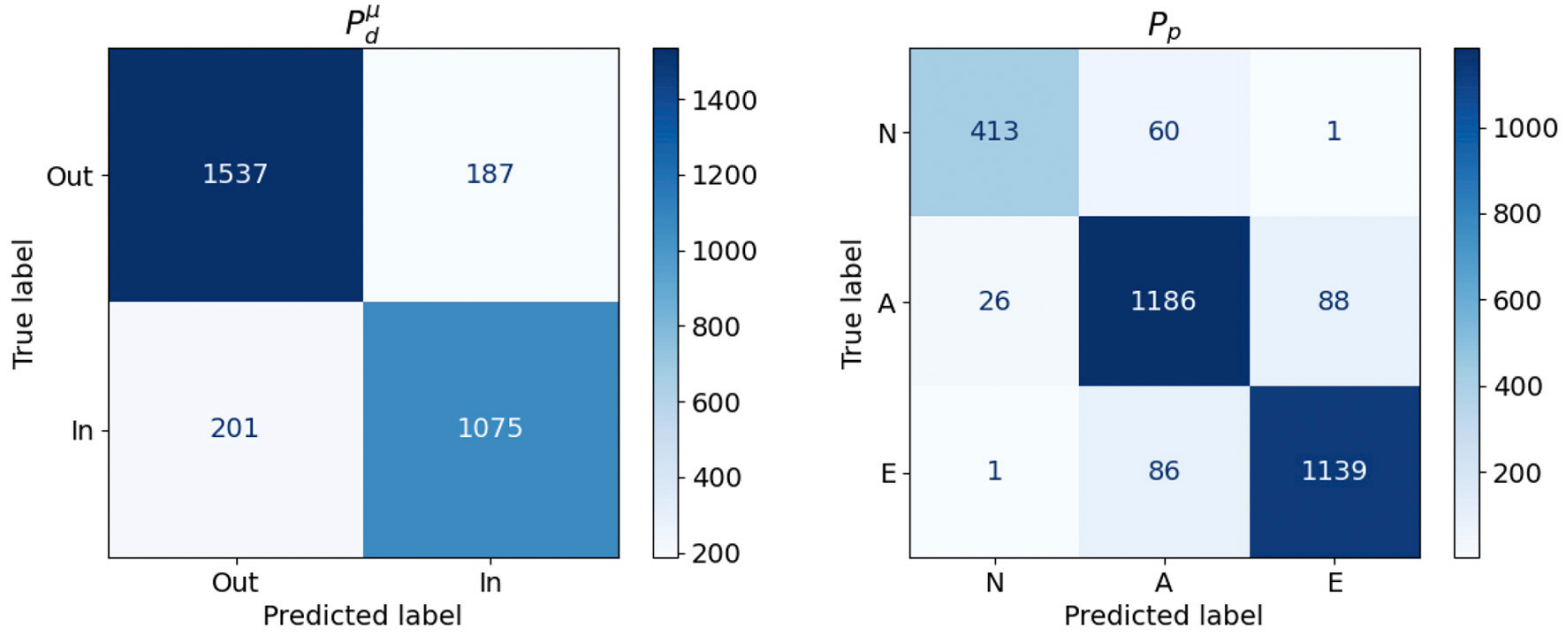
Confusion Matrices obtained for the $P_{d}^{\mu }$ and *P*_*p*_ models.

We can trust $P_{d}^{\mu }$ to build our cohort very efficiently, but at the risk of including some aortas that would not pass the actual test. If we want to prevent this, we will need to evaluate $A_{DD}^{\mu }$ on the aortas accepted by $P_{d}^{\mu }$. If this is the case, the relevant indicator from the efficiency perspective is the sensitivity of $P_{d}^{\mu }$ (ratio of correctly accepted aortas with respect to the total number of accepted aortas). An assessment of the sensitivity of the model was performed using the samples generated with the uniform distribution (size *K*_0_ = 3, 000), a dataset that is different to the one used in the training process. The resulting confusion matrix is presented in Figure 8 (left). It shows that the sensitivity is 0.884, meaning that only 11.6% of the aortas evaluated by $A_{DD}^{\mu }$ will be discarded after using $P_{d}^{\mu }$. Note that, even though the number of false negatives is not relevant from the efficiency point of view –rejected aortas will not lead to any evaluation of the acceptance function–, they lead to a bias in the resulting cohort; the aortas that result in false negatives will not be represented in any cohort that has been generated with a surrogate acceptance function. Thus, this possible bias has to be taken into account if the statistical properties of the resulting cohort is very relevant in our study. In our case, for $P_{d}^{\mu }$, the aortas wrongly rejected represent about a 6% of the total sample of size 3,000 and nearly a 16% of the aortas that should be accepted.

**Figure 9:**
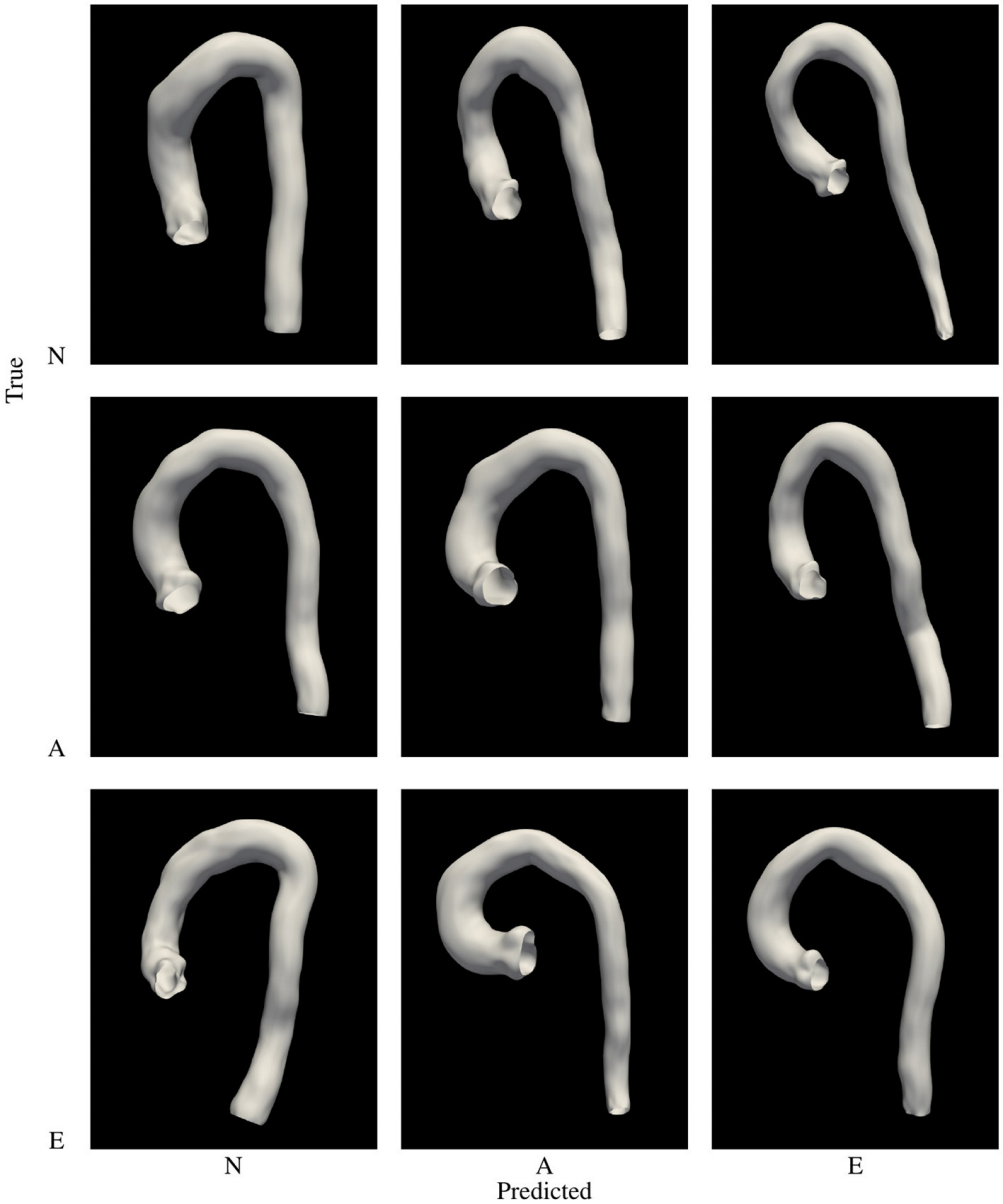
Graphic representation of the confusion matrix of *P*_*p*_. Examples of aortas of phenotype *N*, *A* and *E* row-wise, with the phenotype predicted column-wise arranged.

The second classifier, *P*_*p*_, aims to learn a function able to classify, in the PCA feature space, the three phenotypes used in the clinically-driven cohort generation experiments. A SVC was trained using the set of 3,000 bootstrapped aortas, since this set represents properly the considered phenotype. The best accuracy for this model was 0.92, obtained during 5 fold cross validation using a linear kernel. Again, we tested the performance of *P*_*p*_ by applying the model to the sample of 3,000 aortas generated with a uniform distribution, which is different to the one used during the training process. Figure 8 (right) shows the confusion matrix obtained during this evaluation. We provide a graphical representation of the confusion matrix for *P*_*p*_ in Figure 9. The figure shows an example of an aorta for each one of the scenarios described by that confusion matrix. If our goal is to generate a cohort of only one class, either *N*, *A* or *E*, then the corresponding column of the matrix throws information about the resulting efficiency. For phenotype *N*, we can see that the efficiency (sensitivity) in the test sample is nearly 94% and that the prediction of type *E* presents an efficiency of 93%. On the lower side, phenotype *A* has a ratio of true positives of 89%, leading to the rejection of 11% of the generated geometries. Regarding false negatives, it is noteworthy that class *N* is the one that has higher ratio of improperly rejected aortas, with nearly a 13%.

**Figure 10:**
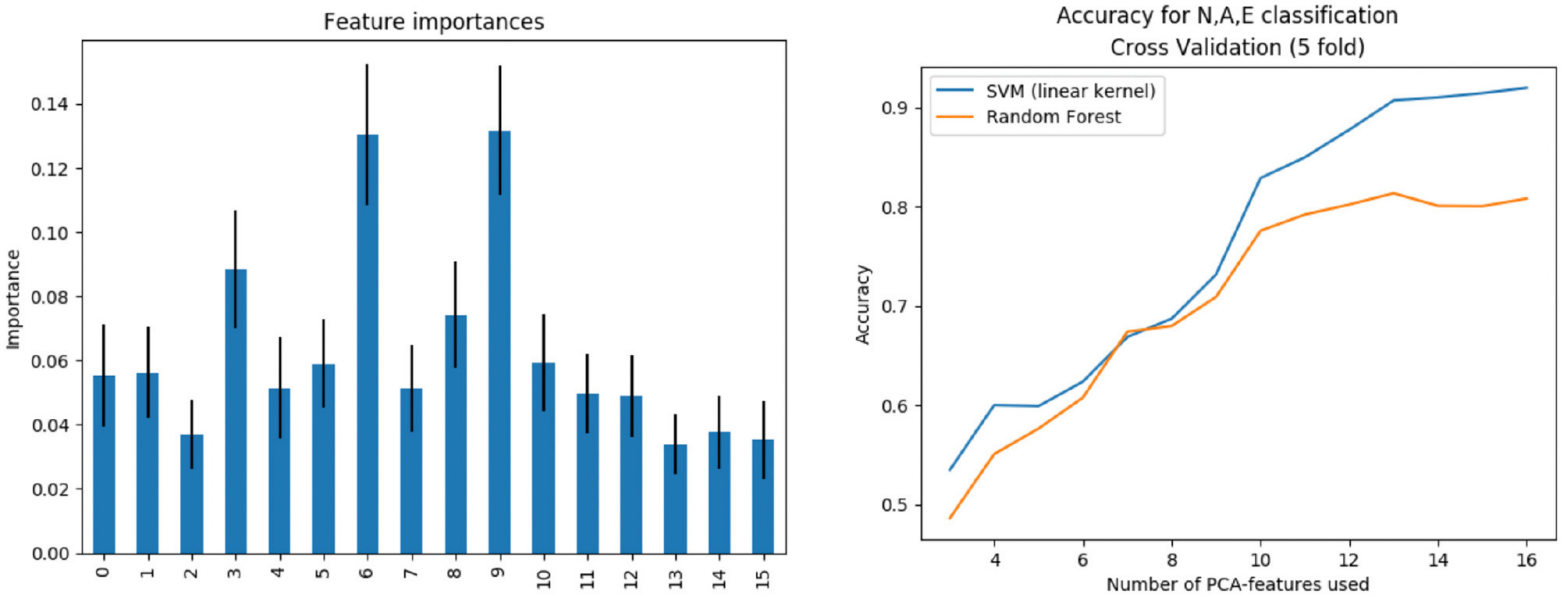
Left: Features importance. Right: Subpopulation classification accuracy as the number of features increases.

Training prediction models opens the possibility of assessing which features are the most relevant for the particular problem we are facing. In the case of the phenotype classifier, *P*_*p*_, we have performed an analysis of the importance of each feature in the decision process. Feature importance has been provided by training a Random Forest model and computing the mean decrease in impurity within each subtree. Figure 10 (left) shows the importance obtained for each feature in the classification problem. As expected, the most important features are those related to the biomarkers involved in the classes definition. For example an inspection of the effect of feature nine on the anatomy shows that the associated deformation mode has a big impact on the Sinuses of Valsalva radius (SoV), which is related to the definition of all three phenotypes.

The training was performed not only for the complete feature vector of dimension 16, but also for the first *n* = 3, 4, …, 16 components, sorted by importance. Figure 10 (right) shows the evolution of accuracy –obtained during the cross validation process– of *P*_*p*_ as the number of features increases, using both the SVM (linear kernel) and the random forest model. Results indicate that, in order to properly separate the three classes, at least 14 features are needed.

In summary, the SVM models are very useful to obtain the decision boundaries of the populations under study, and the data augmentation techniques can take advantage of this ability. The straightforward application is the use of classification models as fast rejection sampling mechanisms in the PCA space, in order to improve the accuracy of the data augmentation technique used at lower cost than rejecting samples in the biomarkers space.

## 4. Discussion

In this study we have shown that there is no universal data-driven cohort generation method, but that the right election highly depends on the purpose of the study. Next, we discuss how the different methods assessed in this paper can be useful according to the needs of the reader. A summary of our findings can also be found in Table 6.

**Table 6:** A summary of the conclusions that can be obtained from the results of the study presented in this paper.

**Method**	**Remarks**	**Sampling scenarios**
Bootstrapping	Well suited when there is no prior knowledge over the variables defining the cohort. It preserves statistical properties of the original sample.	Appropriate if the statistical properties of the resulting sample are relevant, e.g., when the goal is to perform an *in-silico* trial.
Gaussian distribution sampling	Results comparable to bootstrapping in both data-driven and clinically-driven scenarios. It is the most consistent sampling method across the experiments.	Gaussian distribution sampling can provide denser sampling of the tails than bootstrapping, specially if the reference cohort is small. Anatomies far from the mean in feature space can still be underrepresented, leading to unbalanced training sets for Machine Learning models. Assuming normality can bias the sample if the underlying distribution is not Gaussian.
Uniform distribution sampling	Increases the variance of the synthetic sample more than any other method. Oversamples the tails of the observed distribution where less plausible individuals can be found, leading to low efficiency for data-driven acceptance functions.	Not suitable for reproducing the original statistical properties observed in the reference cohort, it can provide better balanced training sets for Machine Learning models.
Generative Adversarial Networks	Achieves good results in the clinically driven scenario, with high efficiency and variance. Sensitive to training set size. Worse results than probability distribution methods if the reference cohort is small. The sample variance was substantially reduced in our data-driven experiments with 26 aortas.	The limitation of the sample size must be taken into account for the sampling scenario. Statistical properties of the original sample can be lost, specially with small reference cohorts.
Machine Learning surrogates	Combined with a sampling method, they can be used to reduce the number of evaluations of acceptance functions. It still requires building a starting sample to be used as training set.	Statistical properties of the resulting cohort depend on the sampling method. High rates of false negatives can bias the sample by reducing its density in certain regions of the space of aortas.

*For each of the techniques evaluated, we present the main remarks and discuss possible strengths and limitations under different sampling scenarios*.

If the goal is to reproduce the existing sample, in what we call data-driven cohort generation then bootstrapping yields trustworthy results. Gaussian sampling achieves similar results. Nonetheless, for some particular biomarkers it leads to longer tails than bootstrapping, while for others to shorter ones. In conclusion, if the actual distribution of the biomarkers is unknown, this can not be assured. On the other hand, the uniform sampling can be well suited if the goal is to obtain the maximum variability, as in machine learning scenarios. The GAN sampling achieved the best acceptance efficiency in most of the criteria measured in this work. However, statistical conclusions should be drawn carefully since there is no guarantee of preserving the underlying probability distributions. On the contrary, bootstrapping and Gaussian have proven to be robust with more moderate values of efficiency. The use of non-linear methods to predict the anatomical or functional phenotype of interest from a compact PCA representation is the most efficient method to generate virtual cohorts, but at the cost of losing statistical characteristics that will be better preserved with bootstrapping.

Over the last years, virtual populations have been built for a variety of applications in the area of cardiac modeling. Britton et al. (2013) generate a population of 10,000 ionic cellular models by varying randomly a specific set of parameters, as in bootstrapping method, to study the variability in cardiac cellular electrophysiology. Haidar et al. (2013) apply Markov Chain Monte Carlo methodology to generate a cohort of Type 1 Diabetes subjects and test glucose controllers. In a similar approach to our work, Allen et al. (2016) present a strategy to efficiently sample and filter virtual populations of pharmacology models, taking empirical data to build data-driven acceptance criteria. Notwithstanding these works do not focus on the anatomy, they share with our research the essential methodology, especially in the last case.

Other authors do focus in shape generation, mainly with medical image as the source of information. a convolutional neural network segment the left ventricle and left atrium using only synthetic images. Rodero et al. (2021) link the main deformations of a cohort of 19 healthy hearts with the electrophysiological biomarkers acquired via simulation. Instead of randomly sampling, the authors perform a sensitivity analysis over a grid on the PCA space, formed by the 9 main modes of variability (covering 90% of variation). They validate the synthetic cohort comparing the obtained biomakers with distributions from literature. Related to thoracic aorta cohorts, in Liang et al. (2017) use 25 geometries of ascending aortic aneurysm to generate a synthetic cohort of 729 shapes in order to asses aneurysm rupture risk using an SVM. They use uniform sampling in the intervals [μ−2σ, μ+2σ] for the first three modes of variation of the PCA. In each one of these works, a particular sampling methodology is chosen, according to the goals of their research. In our paper, we do not focus on a particular clinical outcome but on the methodology itself, providing a systematic comparison of some of these methods.

A common question in any computational anatomy study is the ability of our parametric space to capture the desired real clinical variability. This is pretty difficult to ascertain, and a surrogate metric is the compactness of the PCA basis. In this respect, our study required 16 modes to capture 90% of the variance from a sample of diseased aortas presenting ascending aortic aneurysm. A healthy subset of aortas, by Casciaro, required only 6 modes to capture 84%, and another congenital set of aortas, required 19 modes to capture 90%. In Bruse et al. (2017), thoracic aorta geometry is encoded in a PCA space to solve a classification problem by means of hierarchical clustering. They retain the 19 modes of deformation covering 90% of the variance. Liang et al. (2017) cover 80% of variance with the first three modes of deformation. Our results thus fit within the range of variability seen in previous results.

Clinically-driven generation have proven to be much difficult to achieve. The efficiency of the generation of the synthetic cohort has considerable dependence on the acceptance criteria. Our results show this in the low efficiency achieved for aortas of type *A*, which contrasts with the high efficiency obtained with phenotype *N*. In the work by Thamsen et al. (2021), they achieved an efficiency below 0.27 using a Gaussian distribution. They generate a first synthetic cohort of more than 10,000 individuals and apply what they call a stepwise filtering to limit the cohort to aortas suffering from coarctation.

We have seen that classical statistical methods in many cases obtained considerably lower values of efficiency than the GAN, which outperformed the rest of the methods in both, variance and acceptance. This notable increase in the throughput of the GAN is likely to be related with the increase in the training set. In the data-driven scenario, the training set was formed by 26 samples, while in the clinically-driven, initial synthetic cohorts were much larger (between 330 and 1,000 cases). It is also worth to mention that Gaussian achieved results noticeably better than bootstrap and Uniform. This is partly explained by the fact that multivariate Gaussian distribution accounts for the co-variance of the cohort, what makes the drawn samples scatter around the mean and be mainly distributed in the main axes of variation. This is, in general, not true for uniform distribution. Otherwise, the bootstrapping method has a particular limitation in our case; each reference cohort $C_{0}^{X}$, was extracted from an already synthetic cohort, *C*_*B*_ also generated by bootstrapping from the cohort of real aortas, with size 26. This reduces the set of possible values from which to sample when drawing each coordinate of the feature vector. In any case, the efficiency of the sampling methods for the clinically-driven criteria suggested that there is an overlap in the PCA space between the phenotypes *N* and *A*, and phenotypes *A* and *E*.

The evaluation of the acceptance functions A can require a non-negligible amount of computation. Any vector a, has to be translated from the feature space to the biomarkers space to take the decision, being this computation of the biomarkers part of the evaluation of A. In the cases considered here, where only distances in the anatomy are involved, this process requires evaluations of the polynomials that describe the aorta wall. However, biomarkers derived from hemodynamics or from the cardiac function (Liang et al., 2017; Rodero et al., 2021; Thamsen et al., 2021), require the simulation of the process of interest to obtain the involved biomarkers. Even with low resolution models, this process can require a computation time in the range of minutes to hours on a modern workstation. Machine learning and deep neural networks are already being used to accelerate different processes related to simulation of hemodynamics in the aorta or perform diagnosis (Xiao et al., 2016; Liang et al., 2018, 2020; Feiger et al., 2020). We show that, in the generation of virtual patients cohorts, machine learning can replace the evaluation of acceptance functions with high accuracy. We choose to use SVM bacause they are known to be capable of avoid over-fitting in situations where reduced size dataset are available. This means that this strategy could be used without requiring thousands of samples as used in this work, what makes it feasible for simulation-based clinical criteria.

This last point, however, has to be taken into account when using machine learning surrogates to estimate the acceptance functions. In order to fit the model, a training set still needs to be built. While in models such as SVM the required dataset can be relatively small, for GAN and other network-based models are more sensitive to this limitation, as we have seen in the poor performance achieved by the GAN when trained with the original cohort of size 26. Thus, a first cohort generation task has to be completed using the original acceptance functions, no matter how expensive they are. This effort, however, can later pay off by including the training set in the final cohort.

Another limitation of our study is the size of the original sample, with a total of 26 aortas. This limitation, however, also underpins the fact that cohort generation can be addressed even without having large reference datasets. Indeed, the original sample only had 3 aortas of class *N*, and we conjecture that it is feasible to generate phenotypes that are absent from the reference cohort provided that the anatomy can be properly described by the resulting PCA representation.

Among the possible future extensions to our work we consider the addition of Markov Chain Monte Carlo methods to the set of sampling strategies. All the experiments have been performed using a reduces sample of aortas with the same pathology. The proposed methodology could be applied to a larger, perhaps more heterogeneous, reference dataset. This could be of special interest to better assess the performance of GAN in the data-driven experiments. Also, we would like to validate our hypothesis that it is possible to generate clinically-driven cohorts that are not present at all in the reference dataset.

## 5. Conclusions

The generation of synthetic cohorts of patients is a methodology of increasing utility in cardiovascular modeling. In this paper, we have addressed some of the problems faced by the generation of clinically meaningful virtual cohorts. Using the case of aorta cohort synthesis, we have performed a systematic evaluation of sampling methods that are commonly used in Statistical Shape Modeling. According to our experiments, the sampling strategy and the verification of the generated cases can have a great impact on the efficiency of the process and on the quality of the resulting cohort. We identify several scenarios and discuss the quality of the results of the assessed methodologies in each case. In addition, we propose the use of machine learning models to accelerate the cohort generation.

As simulation models in physiology increase their quality, and the application of machine learning models become ubiquitous, the use of virtual cohorts will become more frequent in therapy design, patient stratification or *in-silico* trials. The results of this paper can guide other authors in the process of reliably building synthetic populations.

## Data Availability Statement

The dataset of 3000 bootstraped aortas generated for this study can be found in the CoMMLab Web page at https://commlab.uv.es/repository/.

## Author Contributions

PR, ML, and IG-F developed the geometric representation of the aortas. ML and FM-G investigated the machine learning models. PR, ML, PL, and IG-F designed the experiments. PR, ML, DS, and FM-G performed the experiments. PR, ML, and DS compiled and analyzed the data. PR, ML, PL, RS, FM-G, and IG-F interpreted the results. PR and IG-F developed the formal analysis. IG-F and RS provided the real aorta anatomies. All authors contributed to the manuscript and approved the submitted version.

## Conflict of Interest

The authors declare that the research was conducted in the absence of any commercial or financial relationships that could be construed as a potential conflict of interest.

## Publisher's Note

All claims expressed in this article are solely those of the authors and do not necessarily represent those of their affiliated organizations, or those of the publisher, the editors and the reviewers. Any product that may be evaluated in this article, or claim that may be made by its manufacturer, is not guaranteed or endorsed by the publisher.

## Footnotes

^1^If unimodality cannot be assumed, then the same interval contains, at least, 91% of the probability density of the distribution.
